# Identification of Six Diagnostic Biomarkers for Chronic Lymphocytic Leukemia Based on Machine Learning Algorithms

**DOI:** 10.1155/2022/3652107

**Published:** 2022-11-24

**Authors:** Yidong Zhu, Xinjin Gan, Ruoyan Qin, Zhikang Lin

**Affiliations:** ^1^Department of Traditional Chinese Medicine, Shanghai Tenth People's Hospital, Tongji University School of Medicine, Shanghai 200072, China; ^2^Department of Hematology, Longhua Hospital, Shanghai University of Traditional Chinese Medicine, Shanghai 200032, China; ^3^Department of Oncology, Longhua Hospital, Shanghai University of Traditional Chinese Medicine, Shanghai 200032, China

## Abstract

**Background:**

Chronic lymphocytic leukemia (CLL) is the most common type of leukemia in adults. Thus, novel reliable biomarkers need to be further explored to increase diagnostic, therapeutic, and prognostic effectiveness.

**Methods:**

Six datasets containing CLL and control samples were downloaded from the Gene Expression Omnibus database. Differential gene expression analysis, weighted gene coexpression network analysis (WGCNA), and the least absolute shrinkage and selection operator (LASSO) regression were applied to identify potential diagnostic biomarkers for CLL using R software. The diagnostic performance of the hub genes was then measured by the receiver operating characteristic (ROC) curve analysis. Functional analysis was implemented to uncover the underlying mechanisms. Additionally, correlation analysis was performed to assess the relationship between the hub genes and immunity characteristics.

**Results:**

A total number of 47 differentially expressed genes (DEGs) and 25 candidate hub genes were extracted through differential gene expression analysis and WGCNA, respectively. Based on the 14 overlapped genes between the DEGs and the candidate hub genes, LASSO regression analysis was used, which identified a final number of six hub genes as potential biomarkers for CLL*: ABCA6, CCDC88A*, *PMEPA1*, *EBF1*, *FILIP1L*, and *TEAD2*. The ROC curves of the six genes showed reliable predictive ability in the training and validation cohorts, with all area under the curve (AUC) values over 0.80. Functional analysis revealed an abnormal immune status in the CLL patients. A significant correlation was found between the hub genes and the immune-related pathways, indicating a possible tight connection between the hub genes and tumor immunity in CLL.

**Conclusion:**

This study was based on machine learning algorithms, and we identified six genes that could be possible CLL markers, which may be involved in CLL pathogenesis and progression through immune-related signal pathways.

## 1. Introduction

Chronic lymphocytic leukemia (CLL) is a hematopoietic malignancy characterized by the clonal accumulation of mature B lymphocytes in the peripheral blood, bone marrow, and lymphoid tissues [1]. It was estimated that 20,160 new cases would be diagnosed, and 4,410 cases of death of this disease would occur only in the United States in 2022 [2]. Notably, most newly diagnosed patients present only with asymptomatic peripheral blood lymphocytosis or leukocytosis [3]. Currently, the diagnosis of CLL is based mainly on blood counts, blood smears, and immunophenotyping of circulating B lymphocytes [4, 5]. Immunohistologically, the coexpression of CD5 and CD23 on the clonal population of B cells, detected by flow cytometry, can be sued for the diagnosis of most CLL cases [6, 7]. Unfortunately, a risk of misdiagnosis might exist between CLL and other lymphoid malignancies with similar morphological features and CD5 positivity, such as mantle cell lymphoma [8, 9] and atypical lymphoplasmacytic lymphoma [10]. Neither the prognosis nor the treatment regimen of the aforementioned disorders is the same, leading to serious adverse consequences of a misdiagnosis [11–13]. Due to the overlapping immunophenotypes and the possibility of the occurrence of similar histological patterns, lymphomas may resemble CLL. Hence, flow cytometry, immunohistochemistry, and anatomical pathology have particular limitations in the diagnosis of CLL from other entities [14, 15]. Moreover, CLL cases are not uniform in clinical practice, and some exhibit atypical features such as CD5- or CD23-negative [7, 16, 17]. Therefore, novel, more reliable biomarkers are needed to improve the diagnosis of CLL.

Over the past decade, next-generation sequencing technology and microarray analysis have been widely applied as fundamental methods in neoplastic disorders for multiple clinical utilization, including molecular diagnosis and prediction of prognosis [18, 19]. Genome studies have been conducted to explore the transcriptome changes in CLL, offering a novel method for potential markers and therapeutic targets [20, 21]. For instance, the *PTX3* gene was considered a marker associated with CLL disease [22]. Moreover, the oncogene *MSI2* was identified as a differential prediagnostic marker and potential therapeutic target of CLL [23]. In a previous study, miR-15b and miR-195 were reported to have the potential to function as novel and noninvasive biomarkers in the diagnosis and prognosis of patients with B-CLL [24]. However, these studies still have disadvantages as they are mainly single-center with small sample sizes, and their results have not been verified by large-scale clinical application.

It was demonstrated that the metabolic, protein interaction, and gene expression networks in biological environment fit in a scale-free topological distribution [25]. Genes are clustered in the form of a coexpression network, in which the ones connected with more genes are in the core position in modules with high modular identity, which are called hub genes [26, 27]. In previous studies, they have been distinguished by the gene expression difference of samples subjected to differential expression analysis alone [28, 29]. Weighted gene coexpression network analysis (WGCNA) is a systematic biology method which is focused on establishing the correlation patterns among genes across microarray samples and screening out hub genes without subjective judgement [30, 31]. The least absolute shrinkage and selection operator (LASSO) has a strong predictive value and low correlation for the selection of the best features for high-dimensional data and prevent overfitting during modeling [32, 33]. By its combination with the aforementioned bioinformatics analysis, in the present study, we aimed to identify genes as diagnostic biomarkers for CLL, with their differences and correlations. Additionally, we performed functional enrichment analysis to explore the possible mechanisms of their action and interaction.

## 2. Materials and Methods

### 2.1. Data Collection

Six datasets containing CLL and control samples (Table 1), GSE14853, GSE26725, GSE31048, GSE50006, GSE51528, and GSE55288, were downloaded from the Gene Expression Omnibus database (GEO, https://www.ncbi.nlm.nih.gov/geo/). The raw data of all studied datasets were normalized to eliminate batch effects. We merged GSE14853, GSE26725, GSE50006, and GSE55288 as a training cohort for subsequent analysis, whereas GSE31048 and GSE51528 were selected as diverse validation cohorts to verify the result.

### 2.2. Identification of Differentially Expressed Genes (DEGs)

CLL and control samples were subjected to differential expression analysis using the LIMMA package. DEGs had to conform to the criterion of |logFC| > 2, where FC denotes fold change and adjusted *P* < 0.05. The heatmap and volcano plot of the DEGs were visualized by “pheatmap” and “ggplot2” packages.

### 2.3. Construction of a Gene Coexpression Network

WGCNA was performed on the training cohort to construct a gene coexpression network of CLL. Based on a scale-free topology model, we quantified and integrated goodness of fit with mean connectivity to ascertain the optimal soft threshold. Multiple modules were next detected automatically, and the topological overlap measure was computed to estimate the adjacencies and similarities among the different modules by average hierarchical clustering. Then, the topologically similar modules were combined into a new cluster. The Pearson correlation analysis was performed to assess the correlations between the module genes and the clinical characteristics. The module with the highest correlations was selected, and the genes in the obtained module were further assessed by Gene Significance (GS) and Module Membership (MM). The genes with an absolute value of MM of over 0.8 and GS of over 0.5 were considered as candidate hub genes for CLL. These measurements were analyzed and visualized by “LIMMA” and “WGCNA” packages.

### 2.4. Identification and Validation of the Hub Genes

The genes overlapped between the DEGs and the candidate hub genes from WGCNA were aggregated by the “venn” package. LASSO regression was adopted to identify the optimal panel of the final hub genes. Further, receiver operating characteristic (ROC) curves were constructed to investigate the accuracy and specificity of these genes for CLL diagnosis. Furthermore, the diagnostic value of the hub genes was verified in the validation cohorts (GSE31048 and GSE51528) by differential expression analysis and ROC curves. The results of these analyses were visualized by “ggpubr” and “pROC” packages.

### 2.5. Functional Enrichment and Correlation Analyses

To investigate the possible mechanisms, the DEGs were subjected to Gene Ontology (GO) and Kyoto Encyclopedia of Genes and Genome (KEGG) analyses. In addition, single sample gene set enrichment analysis (ssGSEA) was applied to contrast the enrichment levels of the immune-related functions and cells between the CLL and the control samples. The Spearman correlation analysis was implemented to examine comprehensively the relationship between hub genes and immunity characteristics. The data were evaluated and visualized by the “clusterProfiler”, “enrichplot”, “DOSE”, “pheatmap”, “GSVA”, “GSEABase”, “vioplot”, and “tidyverse” packages.

### 2.6. Statistical Analysis

Data analysis was performed using R software (version 4.1.3). Student's *t*-test was conducted to determine the significance of the discrepancy between the CLL and the control samples. Two-sided *P* < 0.05 was considered to indicate a statistically significant difference.

## 3. Results

### 3.1. Identification of DEGs

**Figure 1:**
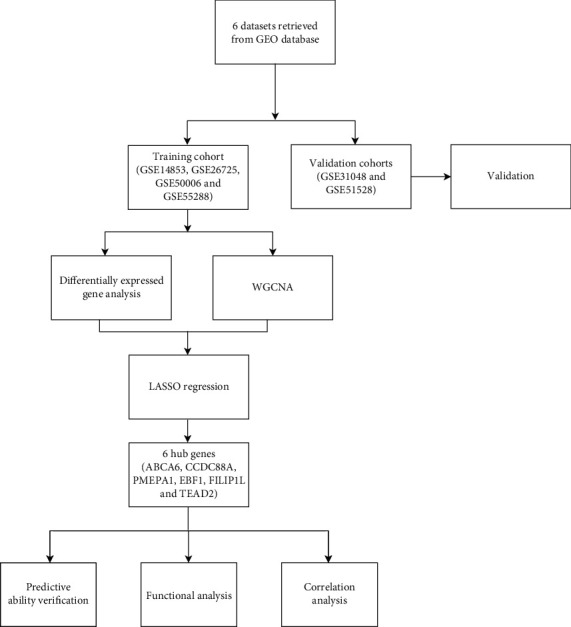
Flowchart of this study.

**Figure 2:**
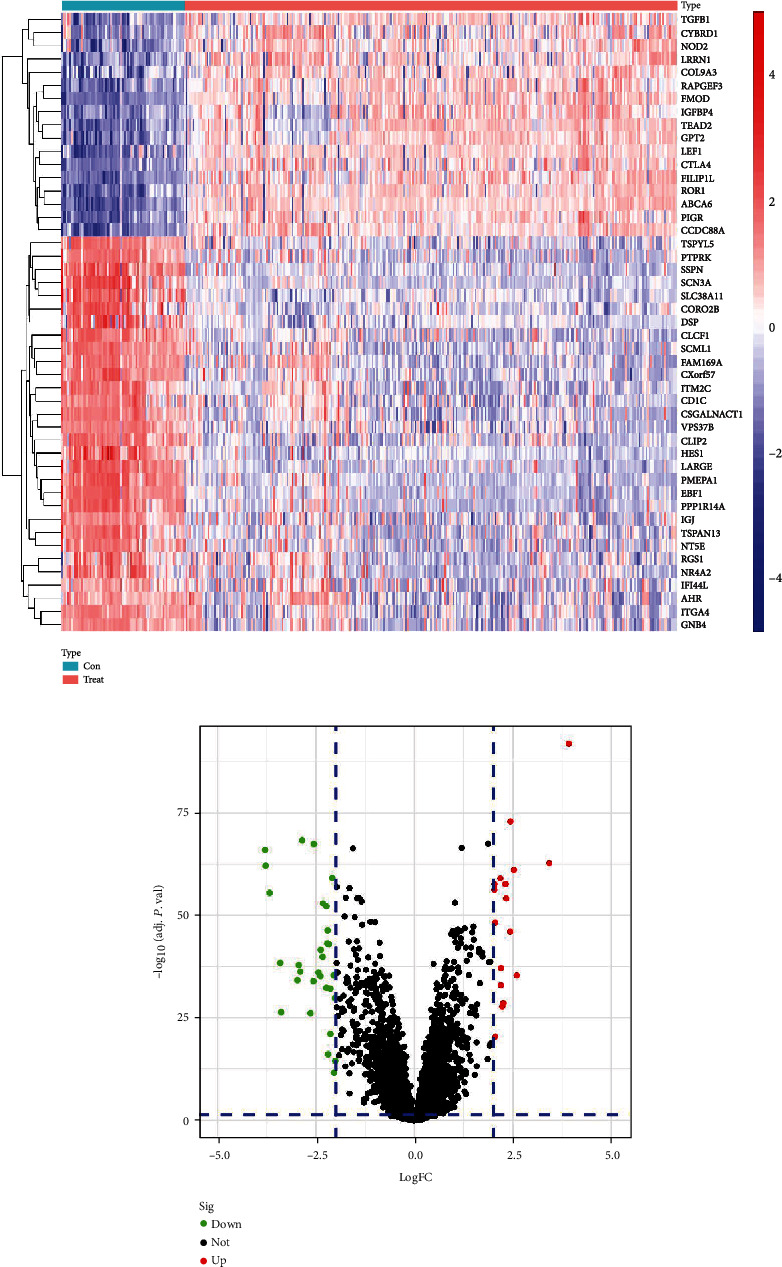
Identification of DEGs between the CLL and the control samples. (a) Heatmap of the DEGs; (b) volcano plot of the DEGs.

The flowchart illustrated in Figure 1 presents the identification and validation of the hub genes as well as the results of the subsequent analyses conducted in the present study. According to the criterion on the training set, 47 DEGs were identified between CLL and control samples, of which 17 were upregulated and 30 were downregulated in CLL (Figures 2(a) and 2(b)).

### 3.2. Construction of a Gene Coexpression Network

**Figure 3:**
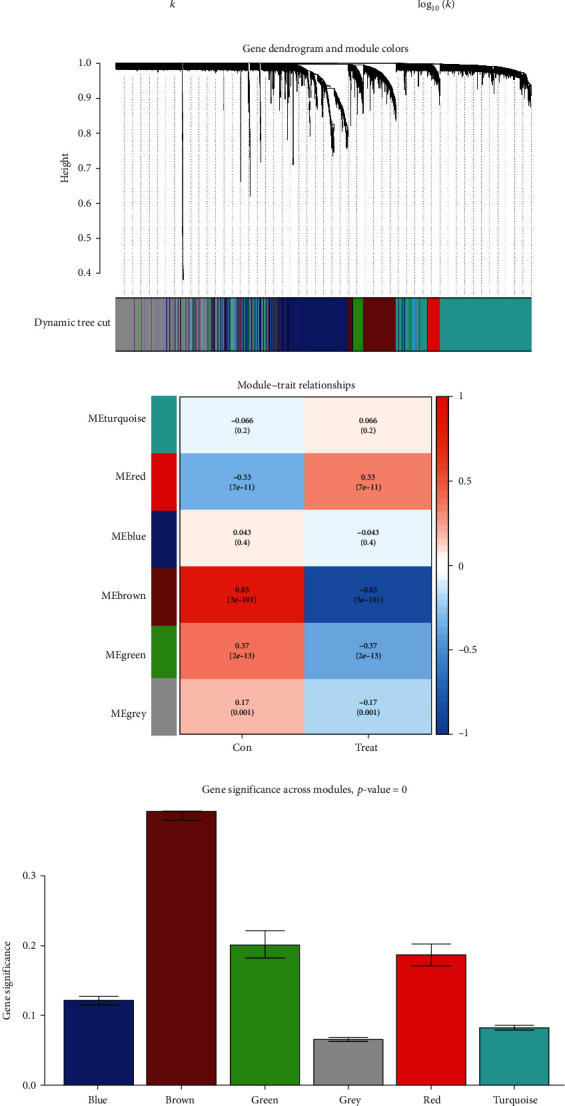
Construction of a gene coexpression network. (a) Analysis of the scale-free network topology for the optimal soft threshold; (b) validation of the optimal soft threshold; (c) WGCNA cluster dendrogram and module assignment; (d) heatmap of the module-trait relationships; (e) histogram of the gene significance across modules; (f) scatterplot of the module membership and gene significance in the brown module.

With the soft threshold of *β* = 5, the network was closer to the real biological network state as it adhered to the power law distribution (Figures 3(a) and 3(b)). A hierarchical clustering analysis based on weighted correlation was further applied. By segmenting the clustering results and merging the similar modules, a total number of six modules were obtained (Figures 3(c)). Of them, the brown module, including 589 genes, showed the strongest correlation with CLL (cor = 0.85, *P* = 3*e* − 101; Figures 3(d) and 3(e)). A tight correlation between GS and MM (cor = 0.95; *P* < 1*e* − 200) was also established. Based on the specified earlier criteria (an absolute value of GS > 0.50 and MM > 0.80), 25 genes in the brown module were identified as candidate hub genes, which were further subjected to subsequent analysis (Figure 3(f)).

### 3.3. Identification of the Hub Genes

**Figure 4:**
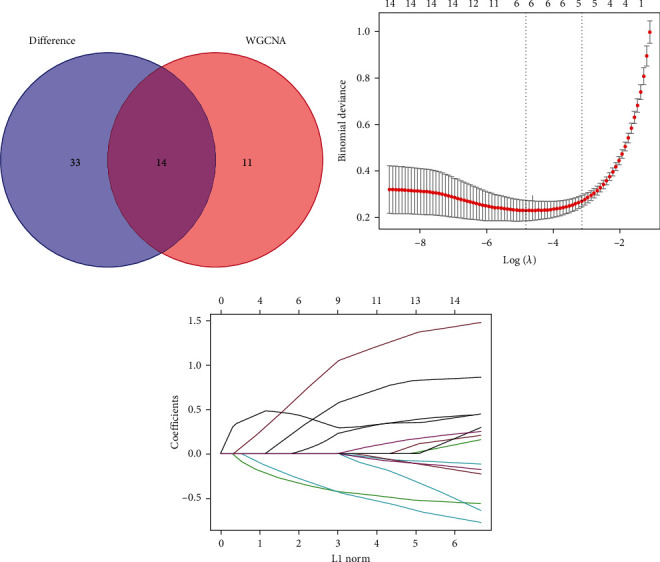
Identification of the hub genes. (a) Venn diagram of the DEGs and the candidate hub genes; (b) and (c) LASSO analysis of the hub genes.

Fourteen overlapped genes were obtained based on the intersections of the DEGs and the candidate hub genes (Figure 4(a)). To prevent overfitting and enhance the accuracy of the diagnostic value, LASSO analysis was utilized to extract the following six finally identified hub genes: *ABCA6*, *CCDC88A*, *PMEPA1*, *EBF1*, *FILIP1L*, and *TEAD2* (Figures 4(b) and 4(c)).

### 3.4. Characterization and Validation of the Hub Gene Expression and Diagnostic Value

**Figure 5:**
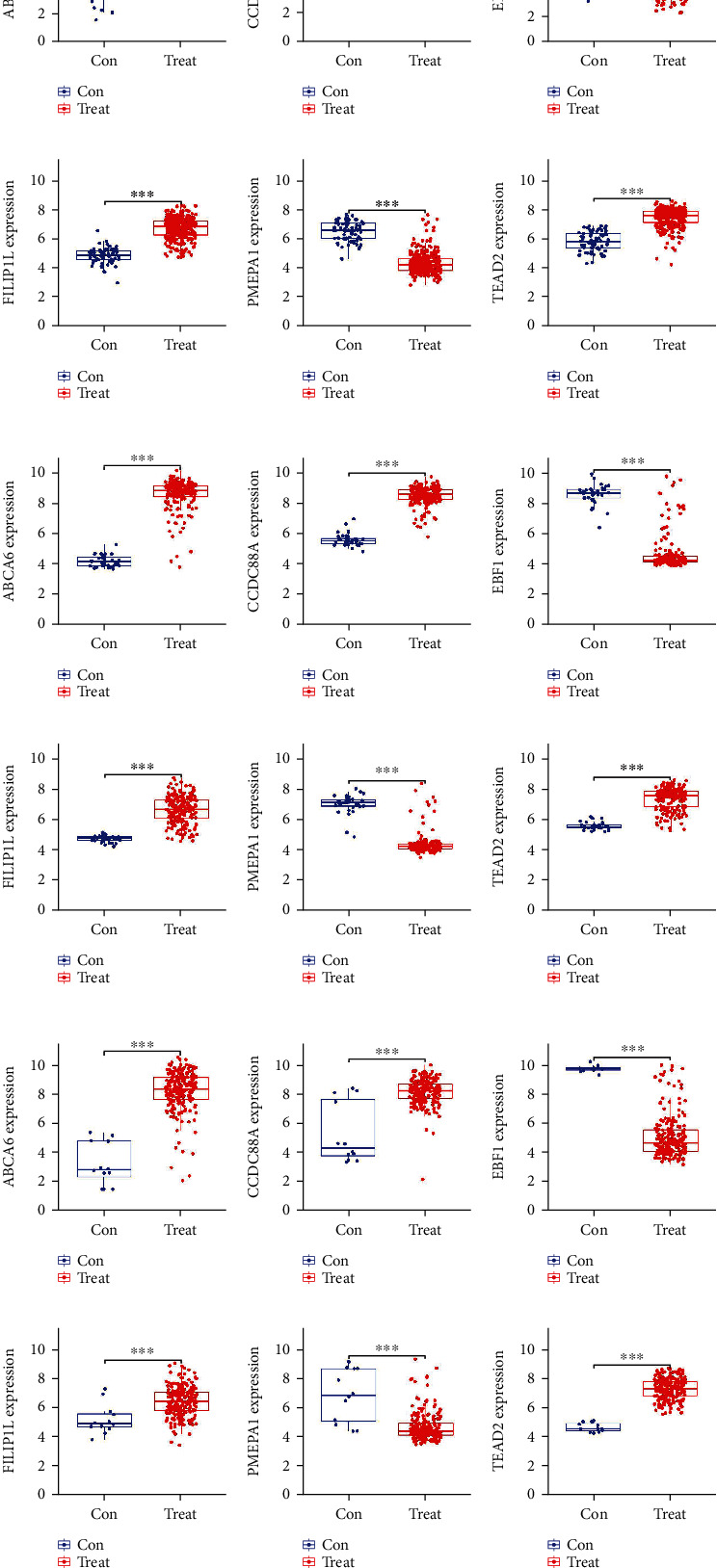
Differential hub gene expression between the CLL and the control samples. (a)–(f) *ABCA6* (a), *CCDC88A* (b), *EBF1* (c), *FILIP1L* (d), *PMEPA1* (e), and *TEAD2* (f) in the training cohort; (g)–(l) *ABCA6* (g), *CCDC88A* (h), *EBF1* (i), *FILIP1L* (j), *PMEPA1* (k), and *TEAD2* (l) in the GSE31048 dataset. (m)–(r) *ABCA6* (m), *CCDC88A* (n), EBF1 (o), *FILIP1L* (p), *PMEPA1* (q), and *TEAD2* (r) in the GSE51528 dataset.

**Figure 6:**
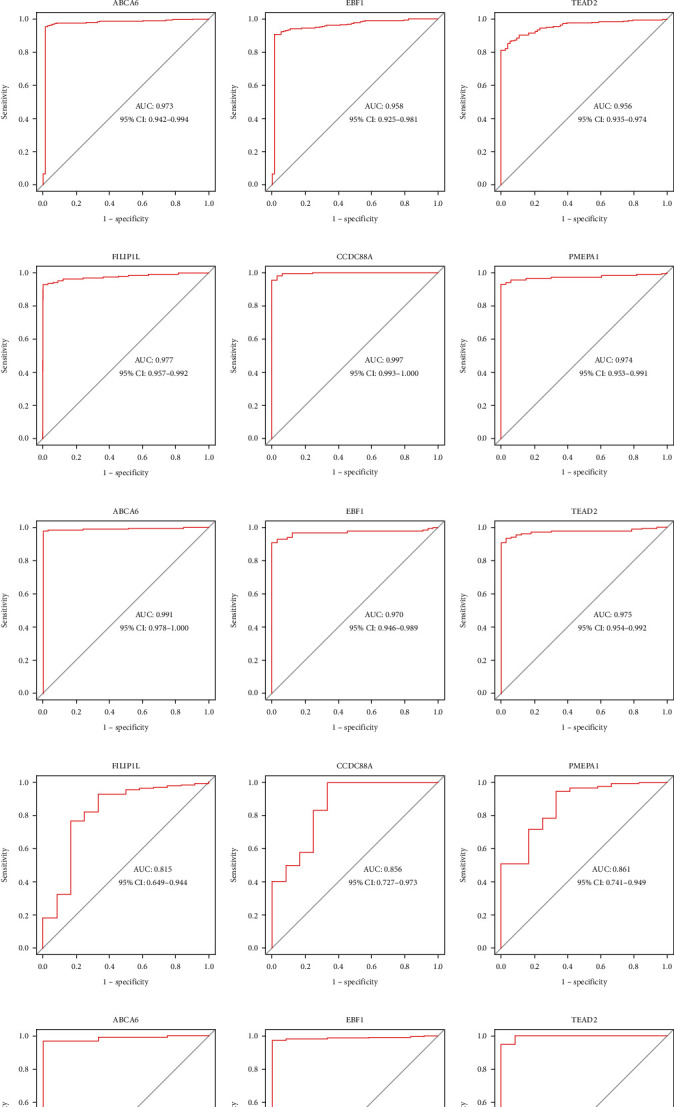
Diagnostic value of the hub genes for CLL. (a)–(f) ROC curves of *FILIP1L* (a), *CCDC88A* (b), *PMEPA1* (c), *ABCA6* (d), *EBF1* (e), and *TEAD2* (f) in the training cohort; (g)–(l) ROC curves of *FILIP1L* (g), *CCDC88A* (h), *PMEPA1* (i), *ABCA6* (j), *EBF1* (k), and TEAD2 (l) in the GSE31048 dataset; (m)–(r) ROC curves of *FILIP1L* (m), *CCDC88A* (n), *PMEPA1* (o), *ABCA6* (p), *EBF1* (q), and *TEAD2* (r) in the GSE51528 dataset.

The boxplots revealed the differential expression of the six hub genes between the CLL and control samples in the training cohort (Figures 5(a)–5(f)). Among them, *ABCA6*, *CCDC88A*, *FILIP1L*, and *TEAD2* were significantly upregulated, whereas *PMEPA1* and *EBF1* were significantly downregulated in the CLL samples. The results were verified in the validation cohorts, and the consistent gene expression patterns were obtained (Figures 5(g)–5(r)). All area under the curve (AUC) values of the six hub genes were over 0.95 in the training cohort (Figures 6(a)–6(f)), showing a satisfactory diagnostic value for CLL. To verify the diagnostic value of the hub genes, we also constructed ROC curves for the validation sets (GSE31048 and GSE51528). The same result was obtained for the GSE31048 dataset, with all AUC values over 0.95 (Figures 6(g)–6(l)). In the GSE51528 dataset, the maximum AUC value was 0.815 for *FILIP1L* (Figure 6(m)). *CCDC88A* (Figure 6(n)) and *PMEPA1* (Figure 6(o)) had AUC values of 0.856 and 0.861, respectively, whereas the other three genes had AUC values over 0.95 (Figures 6(p)–6(r)).

### 3.5. Functional Enrichment and Correlation Analyses

**Figure 7:**
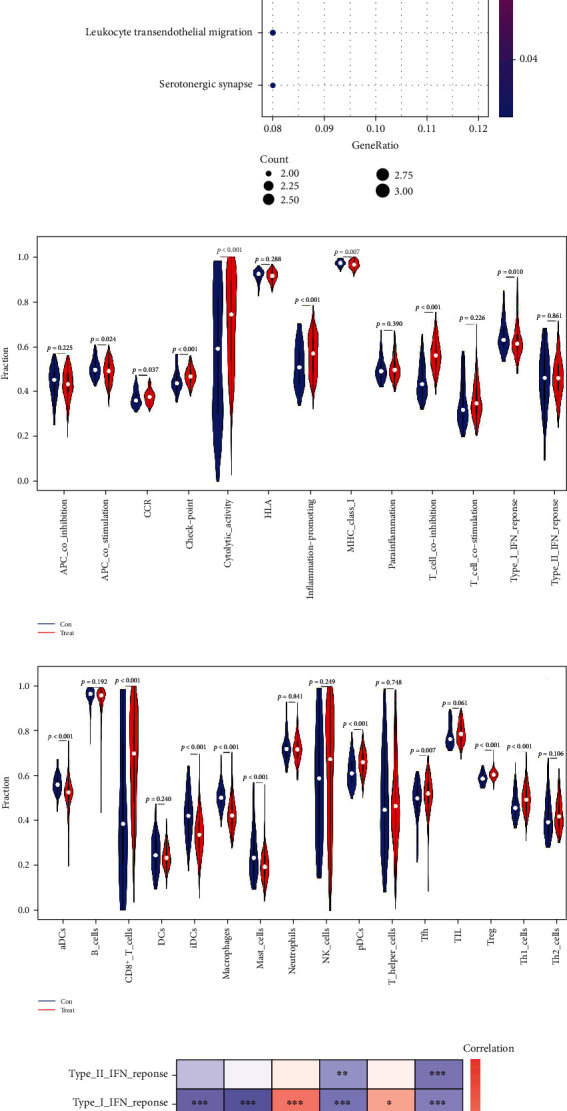
Functional enrichment analysis. (a) The bubble diagram of the gene ontology (GO) analysis; (b) the bubble diagram of the Kyoto Encyclopedia of Genes and Genome (KEGG) enrichment analysis; (c) the violin plot of the immune-related functions; (d) the violin plot of the immune-related cells; (e) the heatmap of the correlations between the hub genes and the immune-related functions; (f) The heatmap of correlations between the hub genes and the immune-related cells.

Functional enrichment analysis was used to establish the possible mechanism involved in the genesis of CLL. GO function analysis results indicated that the biological processes included mainly B-cell proliferation, leukocyte cell-cell adhesion, and positive regulation of leukocyte cell-cell adhesion (Figure 7(a)). The KEGG analysis included predominantly an intestinal immune network for IgA production, ECM-receptor interaction, and hematopoietic cell lineage (Figure 7(b)). The ssGSEA results showed that various immune-related functions and cells had significant differences between the CLL and the control samples (Figures 7(c) and 7(d)). Furthermore, the correlation analysis revealed significant correlations between the hub genes and the immune-related functions and cells (Figures 7(e) and 7(f)), indicating that hub genes may play a crucial role in the pathogenesis and progression of CLL through immune status regulation.

## 4. Discussion

The primary finding of the present study is the successful identification and validation of six diagnostic biomarkers for CLL based on differentially expressed gene analysis, WGCNA, and LASSO regression. The following functional enrichment analysis revealed the crucial impact of immune dysfunction on CLL occurrence and progression. The significant correlations between hub genes and tumor immunity promote the uncovering of the possible mechanism of the involvement of these six genes in the pathogenesis of CLL. The identification of the diagnostic biomarkers might further facilitate the diagnosis of CLL in clinical practice.

In the present study, 47 DEGs and 25 candidate hub genes were extracted through differentially expressed gene analysis and WGCNA, respectively. Subsequently, LASSO regression analysis was used to identify the six final hub genes from the fourteen intersecting genes between the DEGs and the candidate hub genes. The gene expression patterns in the training cohort and different validation cohorts were consistent, with a statistically significant difference between the CLL and the control samples. Nevertheless, the values of control samples in the GSE51528 dataset were not the same as those in other cohorts. Unlike other datasets using normal cells isolated from peripheral blood as normal controls, the GSE51528 dataset used normal B-cell subpopulations from tonsils as controls. The expression of the same gene could be different in various tissues, and the AUC value could have differed because of the use of different tissues as controls. Despite this drawback, the AUC values of the six genes were over 0.8. The six newly identified biomarkers still had a good performance in predicting CLL diagnosis even in the tonsils as controls instead of in specimens of peripheral blood. Of the identified hub genes, *ABCA6*, a member of the ATP-binding cassette transporter family, acts as a probable transporter which may play a role in macrophage lipid transport and homeostasis [34, 35]. *PMEPA1* regulates cell proliferation, differentiation, migration, and immunosuppression through the TGF-beta signaling pathway [36, 37]. *EBF1* has been confirmed to be the key pioneer transcription factor of B-cell specification and commitment by poising or activating lineage-specific genes and repressing genes related with alternative cell fates [38–40]. Overexpressed *FILIP1L* was found to modulate the antiangiogenic activity in endothelial cells, leading to inhibition of cell proliferation and migration as well as an increase in apoptosis [41]. *TEAD2* has been found to be relevant to tumor suppression by restricting proliferation and promoting apoptosis through the Hippo signaling pathway [42, 43]. *CCDC88A* serves as a nonreceptor guanine nucleotide exchange factor which binds to and activates guanine nucleotide-binding protein G (i) alpha subunits, involved in multiple biological processes, such as cell migration and cellular immunity [44–48]. However, the specific role of these six genes in CLL has not been reported yet.

To explore the possible mechanisms by which the hub genes were involved in the nosogenesis of CLL, we performed GO, KEGG, ssGSEA, and correlation analyses. GO function analysis indicated that the biological processes of DEGs were enriched mainly in B-cell activation and proliferation as well as the regulation of leukocyte cell-cell adhesion. KEGG analysis predominantly included immune-associated pathways (e.g., an intestinal immune network for IgA production, ECM-receptor interaction, and leukocyte transendothelial migration). These results suggest that CLL is closely related to tumor immunity. Therefore, ssGSEA was applied to further investigate the differences in the immune cell infiltration between CLL and control samples. We found that T-cell coinhibition was significantly upregulated in the CLL group, which is a vital element contributing to immune function suppression by providing inhibitory signals to activated T cells [49, 50]. Furthermore, various immune-related cells, such as CD8+ T cells and regulatory T cells (Tregs), were significantly more overexpressed in the CLL samples than in the controls. CD8+ T cells exhibited profound functional deficits in the proliferation and cytotoxicity despite an increase in their absolute numbers in the peripheral blood [51, 52]. Treg, which is a subset of CD4+ T cells, was also found to be increased in CLL patients [53–55]. Dysregulation of Tregs leads to an unbalanced immune system and contributes to immune suppression, disease progression, and poor prognosis [56–58]. Coinhibitory molecules expressed by tumor infiltrating T cells and Tregs were previously considered to constitute a pivotal mechanism by promoting tumor immune evasion [59, 60]. The correlations of the hub genes identified here with immune-related functions and cells were also determined, showing significant interactions between the hub genes and tumor immunity. Thus, it is reasonable to assume that the selected genes may modulate the tumor microenvironment and promote tumor immune evasion in CLL through inhibitory immune cells. Serious defects in the immune system and the capacity of leukemic cells to escape immune recognition played an irreplaceable role in the nosogenesis and progression of CLL, which was in agreement with the findings of the present study [1, 61]. Therefore, we speculated that the biomarkers established may be involved in the suppression of the immune response, which supports the proliferation and survival of CLL cell through its immune-related pathways.

This study is not without limitations. First, multiple datasets were retrieved from GEO database, which could have increased the potential heterogeneity due to disparate annotation platforms and clinical covariates of the samples included. The batch effects among datasets could not be completely eliminated. Second, we used public data in our investigation, and thus prospective investigations are needed to validate its predictive power in research with clinical samples. Finally, the underlying molecular mechanisms of the novel biomarkers were not undermined and confirmed by *in vivo* or *in vitro* studies. Hence, the aforementioned deficiencies will be addressed in our further research.

## 5. Conclusion

In conclusion, six genes (*ABCA6*, *CCDC88A*, *PMEPA1*, *EBF1*, *FILIP1L*, and *TEAD2*) for CLL diagnosis were identified by bioinformatics analysis. In addition, the constructed ROC curves confirmed that these genes possessed a good diagnostic value for CLL with high sensitivity and accuracy. Integrated analyses revealed significant interactions between these hub genes and tumor immunity, indicating that the biomarkers may promote CLL cell tumorigenesis and survival via the suppressed immune response through its immune-related pathways. Machine learning algorithms identified that these genes could be possible CLL marker genes, providing the foundation for further experimental studies. However, future research is needed to evaluate the performance of these hub genes and their precise underlying mechanism of action in clinical practice.

## Figures and Tables

**Table 1:** Characteristics of the studied datasets.

GEO series	Control samples	CLL samples	Data type
GSE14853	3	34	Training cohort
GSE26725	5	12	Training cohort
GSE31048	33	188	Validation cohort
GSE50006	32	188	Training cohort
GSE51528	12	217	Validation cohort
GSE55288	33	59	Training cohort

## Data Availability

The datasets used and/or analyzed during the current study are available from the corresponding author on reasonable request.
